# A TaqMan^®^ Assay Allows an Accurate Detection and Quantification of *Fusarium* spp., the Causal Agents of Tomato Wilt and Rot Diseases

**DOI:** 10.3390/biology12020268

**Published:** 2023-02-08

**Authors:** Maria Doroteia Campos, Carla Varanda, Mariana Patanita, Joana Amaro Ribeiro, Catarina Campos, Patrick Materatski, André Albuquerque, Maria do Rosário Félix

**Affiliations:** 1 MED—Mediterranean Institute for Agriculture, Environment and Development & CHANGE—Global Change and Sustainability Institute, Institute for Advanced Studies and Research, Universidade de Évora, Pólo da Mitra, Ap. 94, 7006-554 Évora, Portugal; 2 MED—Mediterranean Institute for Agriculture, Environment and Development & CHANGE—Global Change and Sustainability Institute, Departamento de Fitotecnia, Escola de Ciências e Tecnologia, Universidade de Évora, Pólo da Mitra, Ap. 94, 7006-554 Évora, Portugal

**Keywords:** *Solanum lycopersicum*, *Fusarium* diseases detection, TaqMan^®^ probe assay, qPCR

## Abstract

**Simple Summary:**

In tomato plants, *Fusarium* spp. have been increasingly associated with several wilt and rot diseases that are responsible for severe yield losses. In this context, a molecular-based tool was developed to increase the accuracy of detection and quantification of *Fusarium* spp. genomic DNA (gDNA) in tomato plants and to discriminate *Fusarium* spp. from other fungal species that affect tomato. This assay revealed to be highly specific and sensitive for *Fusarium* species. The used methodology also allowed the establishment of an absolute DNA quantification method. Finally, the effectiveness of the assay was successfully validated with the detection and quantification of *Fusarium* spp. in potentially infected tomato plants from an experimental field and in control plants grown under controlled conditions. The established methodology allows a reliable, sensitive, and reproducible estimation of *Fusarium* accumulation in infected tomato plants, gaining new insights for disease control and providing an additional tool in the screening of resistant plants.

**Abstract:**

In tomato plants, *Fusarium* spp. have been increasingly associated with several wilt and rot diseases that are responsible for severe yield losses. Here, we present a real-time PCR TaqMan^®^ MGB (Minor Groove Binder) assay to detect and discriminate *Fusarium* spp. from other fungal species that affect tomato plants. The methodology used is based on the selective amplification of the internal transcribed spacer (ITS) region of *Fusarium* spp. This assay revealed to be highly specific and sensitive for *Fusarium* species, targeting only the 29 *Fusarium* isolates from the 45 tested isolates associated to tomato diseases. Sensitivity was assessed with serial dilutions of *Fusarium* genomic DNA, with the limit of detection of 3.05 pg. An absolute DNA quantification method was also established, based on the determination of the absolute number of target copies. Finally, the effectiveness of the assay was successfully validated with the detection and quantification of *Fusarium* spp. in potentially infected tomato plants from an experimental field and in control plants grown under controlled conditions. The established methodology allows a reliable, sensitive, and reproducible estimation of *Fusarium* accumulation in infected tomato plants, gaining new insights for disease control and providing an additional tool in the screening of resistant plants.

## 1. Introduction

Tomatoes (*Solanum lycopersicum*), with 187 Mt of production and 5.05 Mha of cultivated area (http://www.fao.org/faostat/en/, accessed on 25 November 2022), are the second most cultivated vegetable crop throughout the world following potatoes [1]. However, they are affected by different pathogens such as fungi, viruses, viroids, oomycetes, bacteria, and nematodes that reduce yield and affect product quality. These pathogen agents cause wilting, leaf spots/blight, fruit spots, and rotting [2].

Among the fungal diseases that affect tomato, we highlight the ones caused by *Fusarium* species. *Fusarium* spp. are ubiquitous soil-borne fungal pathogens which cause destructive vascular wilting, rotting, and damping-off diseases [3,4,5]. As soil-borne pathogens, *Fusarium* species produce persistent resting structures that are able to survive in the soil for long periods of time in the absence of host plants as dormant propagules (chlamydospores), with their germination triggered by the presence of the host’s roots [3,6]. This feature makes eradication quite difficult since chlamydospores may serve as a source of inoculum for infection in subsequent growing seasons [7]. Once the mycelium invades the root cortical cells, the fungi tend to colonize exclusively inside the vessels of xylem, rapidly colonizing the host and producing microconidia, which are transported upwards through the sap stream upon detachment. The characteristic wilt symptoms appear due to vessel blockage triggered by the gathering of fungal hyphae and a combination of the host and pathogen [3,6]. In addition to the losses caused before or during harvest, some *Fusarium* species have the ability to produce mycotoxins that are increasingly becoming a public health concern due to the frequent contamination of various food commodities [8,9].

*Fusarium oxysporum* is an important fungus, known for its phylogenetic diversity [10]. Considering the *F. oxysporum* species, we highlight the two most important formae speciales *F. oxysporum* f. sp. *lycopersici* (FOL) responsible for Fusarium wilt and *F. oxysporum* f. sp. *radicis- lycopersici* (FORL) responsible for Fusarium crown and root rot diseases. Both formae speciales cause extensive production losses in tomato fields and greenhouses and continue to present major challenges for tomato production [3,11]. In addition to *F. oxysporum*, other *Fusarium* species are also known to cause extensive worldwide crop losses such as *Fusarium equiseti*, *Fusarium graminearum*, *Fusarium proliferatum*, *Fusarium solani*, and *Fusarium verticillioides*, all of them associated with tomato wilt [12,13,14,15,16].

The need to increase food production together with the demand to reduce the application of synthetic chemicals that have consequences on increasing the carbon footprint and negative impacts on the environment and human health, led to the search for alternative methods to protect plants against pathogens. Appropriate management practices are invaluable in reducing plant disease losses and have already demonstrated effectiveness in reducing the presence of *Fusarium* species [11,17]. Biological control using antagonistic microorganisms may also constitute an alternative treatment to control plant diseases with quite promising prospects in several plant species, including tomato [10,18,19,20,21]. Moreover, the identification of candidate genes in susceptible and resistant responses may facilitate genetic engineering efforts to incorporate new sources of resistance in tomato plants for protection against pathogens for sustainable plant disease management [4,16]. The use of the highly sensitive real-time quantitative PCR (qPCR) arises as an extremely useful tool for studying various agents of infection in plants, such as fungi, viruses, or bacteria [22,23,24], leading to a better control of diseases and limiting the use of chemical defence strategies.

Given the high incidence of diseases in tomato plants caused by *Fusarium* spp., their consequent negative economic impacts, and the fact that most phytosanitary treatments are based on the application of synthetic fungicides, the establishment of a molecular-based tool that enables their early and accurate detection is of great interest. Furthermore, it will provide an additional tool for the screening of resistant plants. Until now, qPCR has been applied for the detection and quantification of *Fusarium* species individually or targeting few species at the same time [7,25,26]. In the study presented here, a new TaqMan^®^-based qPCR method targeting the *Fusarium* spp.-specific internal transcribed spacer (ITS) region was developed for the simultaneous detection and quantification of a panoply of *Fusarium* species that affect tomato plants. As a proof of principle, the new qPCR assay was used to assess *Fusarium* spp. contamination of tomato field plants and of plants grown under controlled conditions. qPCR combined with the chemistry of TaqMan^®^ MGB probes represents a highly specific and sensitive detection system, even when low amounts of target DNA are present, as in the case of early plant–fungi interactions.

## 2. Materials and Methods

### 2.1. Fungal Isolates

For specificity testing in qPCR assays, potato dextrose agar (PDA) plugs of 29 *Fusarium* spp. isolates and 17 non-*Fusarium* isolates were retrieved from long-term storage at 4 °C, transferred to PDA plates, and maintained at room temperature prior to gDNA extraction. The fungal isolates used in this study belong to the collection of the Mycology Laboratory, Mediterranean Institute for Agriculture, Environment and Development (MED), University of Évora, Portugal, and are listed in Table 1.

### 2.2. Plant Sampling

The tomato plants used in all experiments belong to the UG 29814 variety from United Genetics (Parma, Italy) and have high and intermediate resistance to several diseases, including Fusarium wilt caused by FOL race 3.

For *Fusarium* spp. quantification in tomato tissues, plants from a commercial nursery were planted in a field located in the Ribatejo region (central Portugal) (39.0519608° N, 8.7843653° W). This field has been intensively cropped with tomato plants and known to be infested with Fusarium diseases for many years. At the same time, control tomato plants were planted in plastic pots in a mixture of sand and vermiculite (3:1 ratio), autoclaved, and maintained in a room under controlled temperature (22–25 °C) with a 14 h photoperiod.

A total of 30 tomato plants were randomly collected from the experimental field in mid-July, 12 weeks after plantation. At the same timepoint, 16 tomato control plants were also collected. Plant crowns from all samples were detached and the surfaces were disinfected as previously described [27], ground into powder with liquid nitrogen, and stored at −80 °C until further processing.

### 2.3. gDNA Extraction from Fungal Isolates and Tomato Samples

For each fungal isolate, approximately 100 mg of mycelium from a 2- to 3-week-old PDA culture plate was used for gDNA extraction. gDNA was extracted using the CTAB (hexadecyltrimethylammonium bromide) method [28,29]. For tomato samples, gDNA extraction was performed using DNeasy^®^ Plant Pro Kit (Qiagen, Hilden, Germany) following the manufacturer’s instructions. PCR inhibitors from tomato plants’ gDNA were removed by the OneStep™ PCR Inhibitor Removal Kit (Zymo Research, Freiburg im Breisgau, Germany), also according to the manufacturer’s instructions.

To quantify and assess gDNA purity, the absorbance was evaluated in a Quawell Q9000 micro spectrophotometer (Quawell Technology, Beijing, China). All samples were diluted to a final concentration of 20 ng µL^−1^. gDNA integrity was checked by 0.8% agarose gel electrophoresis.

### 2.4. Design of a Fusarium spp.-Specific qPCR Assay

**Figure 1:**
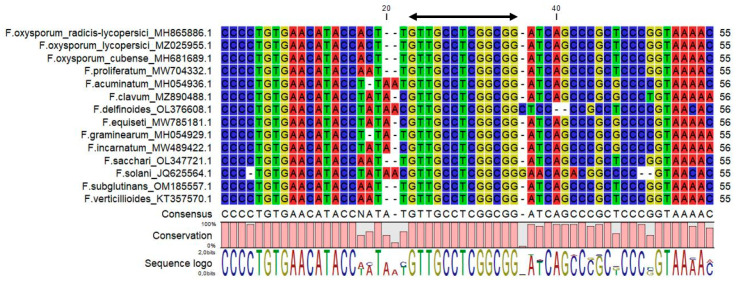
Multiple alignment of a partial sequence of the ribosomal internal transcribed spacer (ITS) region of nuclear rDNA of *Fusarium* species and respective GenBank accession numbers. The location of the probe is indicated with the arrow and corresponds to the position 80 bp from the complete sequence of *Fusarium oxyporum* f. sp. *radicis-lycopersici* (accession number MH865886.1).

Partial sequences of the ITS region from *Fusarium* spp. (including *F. acuminatum*, *F. clavum*, *F. delfinoides*, *F. equiseti*, *F. graminearum*, *F. incarnatum*, *F. oxysporum* f. sp. *cubense, F. oxysporum* f. sp. *lycopersici, F. oxyporum* f. sp. *radicis-lycopersici*, *F. proliferatum*, *F. sacchari*, *F. solani*, *F. subglutinans*, and *F. verticillioides*) (Figure 1), mostly isolated from tomato plants, were retrieved from the National Centre for Biotechnology Information (NCBI) (http://www.ncbi.nlm.nih.gov/ accessed on 6 January 2023). Sequences were aligned using the MUSCLE method [30] integrated in the CLC Genomics Workbench 11 (Qiagen, Hilden, Germany). Nucleotide sequences that showed a specific consensus for *Fusarium* spp. were chosen and used with Primer Express 3.0 software (Applied Biosystems, Foster City, CA, USA), selecting the option MGB TaqMan^®^ probes and the default parameters of the software to design a specific probe (FusProbe: 5′-GTTGCCTCGGCGG-3′) (Figure 1). The 5′ end of the *Fusarium* TaqMan^®^ probe was labelled with 6-FAM (fluorescein), bearing at the 3′ end a nonfluorescent quencher (NFQ) coupled to the Minor Groove Binder moiety (MGB) (Eurogentec, Seraing, Belgium). ITS1 and ITS2 primers (ITS1: 5′-TCCGTAGGTGAACCTGCGG-3′; ITS2: 5′-GCTGCGTTCTTCATCGATGC-3’) [31] were selected as the probe’s flanking primers, generating an amplicon size of 227 bp. To ensure the specificity of the *Fusarium* spp. assay and to demonstrate the suitability of the ITS1-ITS2 region for the design of the specific assay, a bioinformatic analysis was performed, using the correspondent target partial sequence from the ITS region of the *Fusarium* species, as well as from other fungal species previously reported to affect tomato plants in the Mediterranean Basin [1]. A Maximum Likelihood phylogenetic analysis based on the Tamura–Nei model of MEGA 11 software [32] was performed using a bootstrap of 1000 replicates.

### 2.5. Specificity, Sensitivity, and Reliability of the qPCR Assay

All qPCR reactions were performed in 96-well plates in a Line Gene 9600 Plus real-time PCR detection system (BIOER, Hangzhou, China). Reaction mixes contained 100 ng of gDNA as the template, 2× SensiFAST Probe Hi-ROX Kit (Meridian Bioscience, Newtown, OH, USA), 400 nM of each primer, and 100 nM of the probe (Eurogentec, Liège, Belgium) in a total volume of 20 µL. The quantification cycle (Cq) values were obtained for each sample with the following cycling conditions: 20 s at 95 °C for initial denaturation, an amplification program of 40 cycles at 95 °C for 15 s, and 60 °C for 20 s. The fluorescence threshold was manually set to 150. Three technical replicates were considered for each sample. *Fusarium* sp. positive controls and no template controls were included in all plates.

For species specificity validation, a *Fusarium* assay was performed on the gDNA of the 45 described isolates, most of them commonly associated with tomato diseases, including 29 *Fusarium* spp. isolates (Table 1). As a measure of sensitivity and the quantitative range of the developed qPCR procedure, the limit of detection (LOD) was determined. A total of 20 standards were prepared by a two-fold serial dilution of the gDNA of *F. oxysporum* f. sp. *radicis-lycopersici* (2^−1^, 2^−2^,…, 2^−20^, starting with 100 ng) in a background of tomato gDNA from a healthy plant (Table 2). To determine the reliability of the assay, the detection and quantification of *Fusarium* spp. was performed in 30 potentially infested tomato plants from the experimental field and in 16 control plants (for plant sampling details, see Section 2.2) (Table S1).

### 2.6. gDNA Calibrator Plasmid

The method used for the absolute DNA quantification was based on the determination of the absolute number of target copies (TCN) present in each sample, corresponding to haploid genome equivalents [33]. To obtain a specific calibrator plasmid to be used on qPCR, a gDNA region of *F. oxysporum* f. sp. *radicis-lycopersici* that comprised the corresponding TaqMan^®^ target sequence was amplified using the ITS1 and ITS4 primers (ITS1 sequence, see 2.4; ITS4: 5′-TCCTCCGCTTATTGATATGC-3′) [31] by end-point PCR, using the DreamTaq™ DNA polymerase (Thermo Scientific, Waltham, MA, USA) as previously described [34]. The separation of the generated amplicons was performed by agarose gel electrophoresis, and the amplicon with the size of 545 bp was cloned into a pGem^®^-T Easy vector (Promega, Madison, WI, USA) and used to transform *Escherichia coli* JM109 (Promega, Madison, WI, USA) competent cells by standard procedures. Plasmid DNA extraction was performed using GeneJET Plasmid Miniprep Kit (Thermo Scientific, Waltham, MA, USA), and the screening of the selected putative recombinant clones was conducted using EcoRI restriction enzyme (Thermo Scientific, Waltham, MA, USA). Plasmid DNA quantification was determined in a Quawell Q9000 micro spectrophotometer (Quawell Technology, Beijing, China). The sequence of the insert of selected recombinant clones was confirmed by Sanger sequencing (Macrogen, Inc., Madrid, Spain: www.macrogen.com) using the T7 and SP6 universal primers located at the cloning vector (flanking the insert at 5′ and 3′). One bacterial clone harbouring each of the recombinant plasmids was chosen.

A ten-fold dilution series of the selected recombinant plasmid DNA was used to draw a six-point calibration curve in the dynamic range chosen (8 × 10^2^ to 8 × 10^7^ TCN), using the qPCR conditions as described above. qPCR amplification efficiency was calculated using the formula E = (10^(−1/slope)^ − 1) × 100 [35], as well as slope and linearity (regression coefficients, R^2^). *Fusarium* spp. gDNA quantification was carried out by interpolating the samples’ Cq values onto the standard curve.

## 3. Results

### 3.1. Specificity and Sensitivity of the Fusarium spp.-Specific qPCR TaqMan^®^ Assay

**Figure 2:**
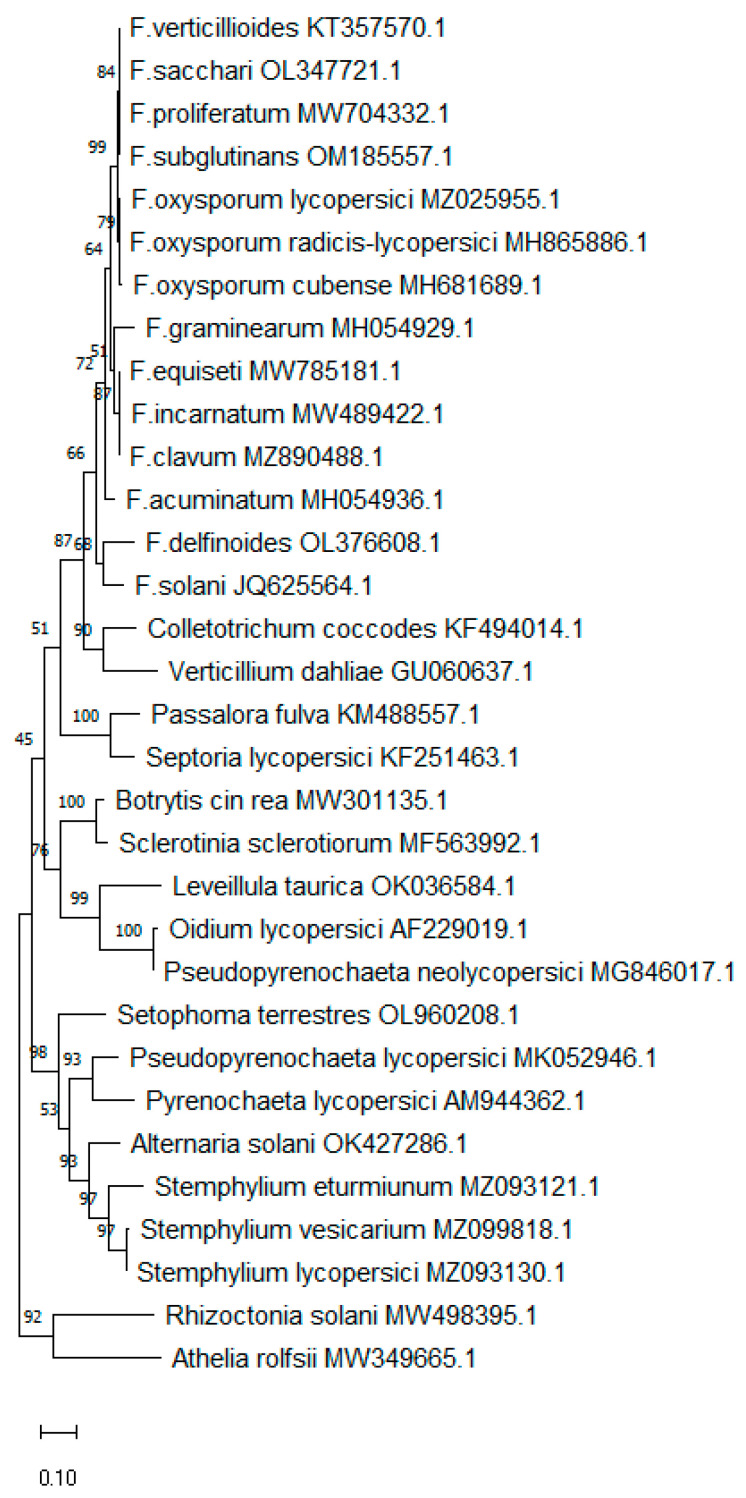
Phylogenetic tree of a partial sequence of the ribosomal internal transcribed spacer (ITS1–ITS2) region of nuclear rDNA by Maximum Likelihood of *Fusarium* spp. and other fungal species that affect tomato plants with respective GenBank accession numbers.

A specific *Fusarium* qPCR assay that specifically targets the ITS region of *Fusarium* spp. in tomato plants was successfully developed, allowing the detection and discrimination of *Fusarium* spp. gDNA from other fungal isolates. The specificity was first demonstrated in silico, performing searches against the NCBI database with the probe targeting *F. oxyporum* f. sp. *radicis-lycopersici*, *F. oxysporum* f. sp. *lycopersici*, *F. solani*, *F. oxysporum* f. sp. *cubense*, *F. incarnatum*, *F. equiseti*, *F. graminearum*, *F. verticillioides*, *F. subglutinans*, *F. proliferatum*, *F. sacchari*, *F. clavum*, *F. delfinoide*, and *F. acuminatum* (Figure 1). The phylogenetic analysis that included *Fusarium* spp. and other fungal species affecting tomato plants in the Mediterranean Basin revealed that the ITS1-ITS2 region forms a *Fusarium* spp. cluster and separates it from the other fungal species (Figure 2 and Figure S1).

To ensure its specificity, the assay was then evaluated experimentally using 16 non-*Fusarium* spp. and 29 *Fusarium* spp. isolates. All *Fusarium* isolates were amplified by the assay, with Cq values varying from 18.42 to 30.32, while no amplification was detected in the remaining isolates (Table 1).

When the sensitivity of the assay (expressed by the LOD) was evaluated, the lowest concentration of gDNA detected corresponded to 3.05 pg of *F. oxysporum* f. sp. *radicis-lycopersici* with a Cq value of 33.42 (± 0.29) (Table 2), achieved in the 2^−15^ dilution. In the remaining points of the dilution series (from 2^−16^ to 2^−20^), no amplification was detected. The LOD, which corresponds to the concentration that can be detected with reasonable certainty (95% probability) in the analytical procedure [36], is defined in qPCR as the spike amount of the target organism in dilution that could be detected in more than 95% of replicates [37]. No interference of host gDNA was observed when a healthy tomato plant was used in the dilutions instead of water (result not shown). The dilution series standard curve showed a linear correlation (R^2^ = 0.993) between Cq and the amount of gDNA template (Figure S2). This study was performed using *F. oxysporum* gDNA due to the prevalence that this species has among the *Fusarium* species that affect tomato plants.

### 3.2. Calibration Curves for Quantification of Fusarium spp. gDNA

**Figure 3:**
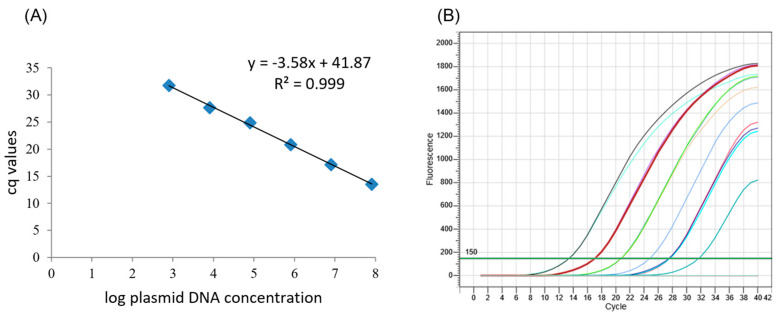
Calibration curve obtained with the *Fusarium* spp.-specific qPCR TaqMan^®^ assay showing copy number of template vs. quantification cycle (Cq). (**A**) Calibration curve constructed based on Cq values obtained from the log of ten-fold dilution series of target plasmid DNA in the dynamic range of 8 × 10^2^ to 8 × 10^7^ target copies. (**B**) Amplification plot where the cycle number increases as target plasmid DNA concentration decreases. R^2^: regression coefficient.

A calibration curve of the 10-fold dilution series of the selected recombinant plasmid DNA was established using the *Fusarium* spp. qPCR assay, which enabled the detection of 8 × 10^2^ TCN, corresponding to a Cq value of 31.73. The calibration curve was characterized by the following parameters: slope (−3.58), Y-intercept (41.87), PCR efficiency (90.2%), and linearity (R^2^ = 0.999) (Figure 3).

The equation of the calibration curve was used to infer the gDNA amount of *Fusarium* species in terms of TCN. In the evaluation of the sensitivity of the assay, the gDNA amount in terms of TCN was then determined in each point of the dilution series (Table 2). A TCN of 229.3 corresponding to 3.05 pg of *F. oxysporum* f. sp. *radicis-lycopersici* was the lowest amount detected by the qPCR assay (Table 2).

### 3.3. Applicability of the Fusarium spp.-Specific qPCR TaqMan^®^ Assay in Tomato Plants

As a proof of concept to evaluate the practical robustness and accuracy of the assay, tomato plants from the experimental field and grown under controlled conditions were sampled, and the *Fusarium* gDNA amount was evaluated. Our results allowed the detection and quantification of *Fusarium* spp. in twelve of the thirty tested samples from the experimental field and in eight of the sixteen samples of the tomato plants grown under controlled conditions (Table S1).

## 4. Discussion

The precise detection and identification of plant-infecting fungi is essential to facilitate effective management of diseases, with DNA-based methods allowing an accurate plant disease diagnosis [38]. Furthermore, when combined with information on plant disease severity, the quantification of fungi offers an additional tool in the screening of resistant plants to fungal diseases [26,39].

In the work presented here, a new *Fusarium* spp.-specific qPCR TaqMan^®^ assay that targets the ITS region was established for the detection and quantification of *Fusarium* spp. in tomato plants, following procedures previously defined for the use of qPCR in microbial diagnostics [37,40]. qPCR combined with the chemistry of TaqMan^®^ MGB probes represents the most specific and sensitive detection system, even in the presence of few amounts of target gDNA [33].

Although the ITS region is quite conserved among fungi, the phylogenetic analysis revealed that the ITS1-ITS2 region was appropriate for the design of the assay since a *Fusarium* spp.-specific cluster was identified (Figure 2), and in silico analysis confirmed the specificity of the assay for *Fusarium* species that might affect tomato plants. In fact, the use of this region for the design of assays benefits from the large number of available sequences in databases, which several studies successfully established [26,41,42]. The specificity of the assay was also verified experimentally using a considerably large group of *Fusarium* and non-*Fusarium* isolates (Table 1). However, a range of Cq values varying from 18.42 to 30.32 was observed among the different *Fusarium* isolates tested (although in 20 of the 29 *Fusarium* isolates, Cq values varied from 18 to 23). This finding might be explained by the existence of single-nucleotide polymorphisms in the *primers* target region that might affect the efficiency of the reaction. It is also possible that the number of rDNA repeats influences the sensitivity of the detection [43]. As further stated by Lavrinienko et al. [44], the rDNA copy number is not necessarily a species-level trait and it can show great intraspecific variability, but still, this locus is not redundant regarding communities’ composition and quantification, requiring a careful interpretation of the results.

The importance of the specificity of the assay is even more relevant in plants growing in natural field conditions that might be infected by multiple pathogens. qPCR has been commonly used for the detection and quantification of *Fusarium* in plants, but often targeting individual *Fusarium* species or few *Fusarium* species at the same time [7,16,25,26,45,46]. However, a single qPCR assay for the broad number of *Fusarium* species that might infect tomato plants, lacking until now, is here applied to this specific group of fungi.

When a calibration curve for the quantification of the *Fusarium* spp. gDNA amount was established, the newly designed assay revealed that E, R^2^, and slope were consistent with the acceptance criteria [47], confirming the accuracy and linear response of the assay over a wide range of dilutions and suggesting the absence of PCR inhibitors. A critical step for an efficient gDNA amplification is to eliminate PCR inhibitors such as the high levels of phenolic compounds present in tomato plants [48] that might affect the sensitivity of the assay [23,49]. PCR inhibitors might be difficult to separate from gDNA during the extraction procedure and can result in false negatives or inaccurate quantification [25]. So, in the studies of the sensitivity of the assay in which the *F. oxysporum* f. sp. *radicis-lycopersici* was diluted it tomato gDNA, as well as in the studies on the applicability of the assay to tomato plants, the use of the DNeasy^®^ Plant Pro Kit combined with the *OneStep*™ PCR Inhibitor Removal Kit was essential for tomato gDNA extraction.

Although FOL and FORL are among the most intensively studied *Fusarium* species that affect tomato plants, responsible for extensive worldwide crop losses [3,39], other *Fusarium* species have demonstrated high incidence, causing severe and mild disease in tomato. *F. solani*, *F. equiseti*, *F. proliferatum*, and *F. verticillioides*, together with *F. oxysporum*, were already associated with rot diseases of tomato [12,13,16]. Akbar et al. [14,15] reported the prevalence of *F*. *equiseti* in tomato plants, which has been increasingly associated with many wilt diseases, followed by *F*. *graminearum*, *F*. *solani*, and *F. acuminatum*. These findings reinforce the idea of the importance of a single assay to target all the *Fusarium* species that might affect tomato.

Unexpectedly, our results allowed the detection and quantification of *Fusarium* spp. in several tomato plants from a commercial nursery grown under controlled conditions. Although these plant varieties present resistance to FOL race 3, these results are most likely explained by the lack of sanitation in the nurseries with the use of infected seeds and/or substrates, as early pathogen detection is crucial to assure the health status of the commercialized plant material [50]. The established assay might be used for the preventive detection of Fusarium diseases in plant material from nurseries, prior to transplantation into production fields.

## 5. Conclusions

Overall, the described *Fusarium* spp. qPCR TaqMan^®^ assay targets a broad range of *Fusarium* species that cause extensive worldwide crop losses of tomato plants, allowing the discrimination of *Fusarium* spp. gDNA from other tomato pathogenic fungi. The assay revealed a high specificity, a high reliability, and a reproducible estimation of *Fusarium* accumulation in infected plants. The high sensitivity makes this methodology an efficient tool for the early diagnosis of the diseases, limiting the use of chemical defence strategies while providing an additional tool in the screening for resistant plants.

## Figures and Tables

**Table 1:** List of isolates of *Fusarium* spp. and other fungal isolates used to test species specificity using the *Fusarium* spp.-specific qPCR assay. Data are expressed as quantification cycle (Cq) values, using 100 ng of gDNA as template. N.D.: not detected.

Species	Isolate Ref.	Cq Value
*F. incarnatum*	M_4F	22.90
*F. oxysporum*	M_45-2	22.86
*F. oxysporum*	M_67	20.48
*F. oxysporum*	M_84	21.53
*F. oxysporum*	M_90	25.05
*F. oxysporum*	M_91	20.31
*F. oxysporum*	M_92	20.26
*F. oxysporum*	M_100	19.29
*F. oxysporum*	A_56	25.08
*F. oxysporum*	A_57	22.76
*F. oxysporum*	A_122	23.57
*F. oxysporum*	A_127a	26.42
*F. oxysporum*	A_127b	30.32
*F. oxysporum*	A_137	25.65
*F. oxysporum*	C_4	18.42
*F. oxysporum* f.sp. *radicis-lycopersici*	C_3	19.20
*F. solani*	M_215c	21.51
*F. verticillioides*	A_118	29.29
*Fusarium nelsonii*	A_82c	20.17
*Fusarium nelsonii*	A_82e	20.17
*Fusarium* sp.	M_62	20.01
*Fusarium* sp.	M_207	20.16
*Fusarium* sp.	M_208	22.19
*Fusarium* sp.	M_209	20.28
*Fusarium* sp.	P_Q7	19.95
*Fusarium* sp.	A_123	24.63
*Fusarium* sp.	A_126	25.91
*Fusarium* sp.	C_1	21.48
*Fusarium* sp.	C_2	21.06
*Alternaria alternata*	A_59	N.D.
*Alternaria alternata*	A_72	N.D.
*Alternaria alternata*	C_6	N.D.
*Alternaria tenuissima*	P_75B	N.D.
*Botrytis cinerea*	A_43	N.D.
*Botrytis cinerea*	A_64	N.D.
*Botrytis cinerea*	A_115	N.D.
*Botrytis cinerea*	A_119	N.D.
*Botrytis cinerea*	A_132	N.D.
*Botrytis cinerea*	C_9	N.D.
*Cladosporium cladosporioides*	P_70D	N.D.
*Colletotrichum* sp.	A_71	N.D.
*Epicoccum nigrum*	P_R46	N.D.
*Phytium* sp.	C_10	N.D.
*Phytophthora* sp.	C_5	N.D.
*Verticillium dahliae*	C_8	N.D.

**Table 2:** Sensitivity of the *Fusarium* spp.-specific qPCR assay using a serial dilution of gDNA from *F. oxyporum* f. sp *radicis-lycopersici*. The lowest amount of detection for both assays can be observed. Data are expressed as quantification cycle (Cq) values and target copy number (TCN). P.C.: positive control; SD: standard deviation; N.D.: not detected.

Dilution	gDNA in PCR (ng)	Cq Value (±SD)	TCN
P.C.	100.00	14.67 (±0.22)	39,606,399.4
2^−1^	50.00	15.53 (±0.27)	22,779,380.0
2^−2^	25.00	16.72 (±0.27)	10,595,944.3
2^−3^	12.50	17.52 (±0.29)	6,333,969.6
2^−4^	6.25	18.57 (±0.27)	3,223,887.6
2^−5^	3.13	19.61 (±0.17)	1,651,494.4
2^−6^	1.56	20.50 (±0.08)	931,695.0
2^−7^	7.81 × 10^−1^	21.73 (±0.26)	422,375.1
2^−8^	3.91 × 10^−1^	23.09 (±0.13)	176,120.6
2^−9^	1.95 × 10^−1^	24.52 (±0.25)	70,205.1
2^−10^	9.77 × 10^−2^	25.82 (±0.19)	30,425.7
2^−11^	4.88 × 10^−2^	26.74 (±0.07)	16,836.7
2^−12^	2.44 × 10^−2^	28.38 (±0.16)	5863.5
2^−13^	1.22 × 10^−2^	30.27 (±0.34)	1738.7
2^−14^	6.10 × 10^−3^	32.52 (±0.59)	409.0
2^−15^	3.05 × 10^−3^	33.42 (±0.29)	229.3
2^−16^	1.53 × 10^−3^	N.D.	N.D.

## Data Availability

Data sharing not applicable.

## Supplementary Materials

The following supporting information can be downloaded at: https://www.mdpi.com/article/10.3390/biology12020268/s1, Figure S1: List of sequences of ITS region of *Fusarium* spp. and other fungal species affecting tomato in the Mediterranean Basin used on the phylogenetic analysis; Figure S2: Sensitivity and linearity of the *Fusarium* spp.-specific qPCR assay. Standard curve constructed based on Cq values obtained from the log of two-fold dilution series of gDNA from *F. oxyporum* f. sp *radicis-lycopersici*. gDNA concentrations vary from 100 ng to 3.05 × 10^−3^ ng; Table S1: Quantification of *Fusarium* spp. in tomato field and control samples using the *Fusarium* spp.-specific qPCR assay. Data are expressed as quantification cycle (Cq) values.
