# Supplementary material for: A TaqMan® Assay Allows an Accurate Detection and Quantification of Fusarium spp., the Causal Agents of Tomato Wilt and Rot Diseases
Source: Biology (Basel). 2023 Feb 8;12(2):268. doi: 10.3390/biology12020268 (PMC9953614; doi:10.3390/biology12020268)
Supplement: Supplementary file 1 [file biology-12-00268-s001.zip › Figure S1.pdf]

```
>F.oxysporum_radicis-lycopersici_MH865886.1
TAACAAGGTTTCCGTTGGTGAACCAGCGGAGGGATCATTACCGAGTTTACAACTCCCAAACCCCTGTGAACATACCACTT
GTTGCCTCGGCGGATCAGCCCGCTCCCGGTAAAACGGGACGGCCCGCCAGAGGACCCCTAAACTCTGTTTCTATATGTAA
CTTCTGAGTAAAACCATAAATAAATCAAAACTTTCAACAACGGATCTCTTGGTTCTGGCATCGATGAAGAACGCAGCAAA
ATGCGATAAGTAATGTGAATTGCAGAATTCAGTGAATCATCGAATCTTTGAACGCACATTGCGCCCGCCAGTATTCTGGC
GGGCATGCCTGTTCGAGCGTCATTTCAACCCTCAAGCACAGCTTGGTGTTGGGACTCGCGTTAATTCGCGTTCCCCAAAT
TGATTGGCGGTCACGTCGAGCTTCCATAGCGTAGTAGTAAAACCCTCGTTACTGGTAATCGTCGCGGCCACGCCGTTAAA
CCCCAACTTCTGAATGTTGACCTCGGATCAGGTAGGAATACCCGCTGAACTTAAGCATATCAATAAGCGGA
>F.oxysporum_lycopersici_MZ025955.1
AACAAGGTCTCCGTTGGTGAACCAGCGGAGGGATCATTACCGAGTTTACAACTCCCAAACCCCTGTGAACATACCACTTG
TTGCCTCGGCGGATCAGCCCGCTCCCGGTAAAACGGGACGGCCCGCCAGAGGACCCCTAAACTCTGTTTCTATATGTAAC
TTCTGAGTAAAACCATAAATAAATCAAAACTTTCAACAACGGATCTCTTGGTTCTGGCATCGATGAAGAACGCAGCAAAA
TGCGATAAGTAATGTGAATTGCAGAATTCAGTGAATCATCGAATCTTTGAACGCACATTGCGCCTGCCTATTCTGGCGGG
CATGCCTGTTCGAGCGTCATTTCAACCCTCAAGCACAGCTTGGTGTTGGGACTCGCGTTAATTCGCGTTCCCCAAATTGA
TTGGCGGTCACGTCGAGCTTCCATAGCGTAGTAGTAAAACCCTCGTTACTGGTAATCGTCGCGGCCACGCCGTTAAACCC
CAACTTCAA
>F.acuminatum_MH054936.1
TTACCGAGTTTACAACTYCCAAACCCCTGTGAACATACCTTAATGTTGCCTCGGCGGATCAGCCCGCGCCCCGTAAAACG
GGACGGCCCGCCAGAGGACCCAAACTCTAATGTTTCTTATTGTAACTTCTGAGTAAAACAAACAAATAAATCAAAACTTT
CAACAACGGATCTCTTGGTTCTGGCATCGATGAAGAACGCAGCAAAATGCGATAAGTAATGTGAATTGCAGAATTCAGTG
AATCATCGAATCTTTGAACGCACATTGCGCCCGCTGGTATTCCGGCGGGCATGCCTGTTCGAGCGTCATTTCAACCCTCA
AGCCCCCGGGTTTGGTGTTGGGGATC
>F.oxysporum_cubense_MH681689.1
TGACCTGCGGAGGGATCATTACCGAGTTTACAACTCCCAAACCCCTGTGAACATACCACTTGTTGCCTCGGCGGATCAGC
CCGCTCCCGGTAAAACGGGACGGCCCGCCAGAGGACCCCTAAACTCTGTTTCTATATGTAACTTCTGAGTAAAACCATAA
ATAAATCAAAACTTTCAACAACGGATCTCTTGGTTCTGGCATCGATGAAGAACGCAGCAAAATGCGATAAGTAATGTGAA
TTGCAGAATTCAGTGAATCATCGAATCTTTGAACGCACATTGCGCCCGCCAGTATTCTGGCGGGCATGCCTGTTCGAGCG
TCATTTCAACCCTCAAGCACAGCTTGGTGTTGGGACTCGCGTTAATTCGCGTTCCTCAAATTGATTGGCGGTCACGTCGA
GCTTCCATAGCGTAGTAGTAAAACCCTCGTTACTGGTAATCGTCGCGGCCACGCCGTTAAACCCCAACTTCTGAATGTTG
ACCTCGGATCAGGTAGGAATACCCGCTGAACTTAAGCATATCA
>F.incarnatum_MW489422.1
CCTGCGGAGGGATCATTACCGAGTTTACAACTCCCAAACCCCTGTGAACATACCTATACGTTGCCTCGGCGGATCAGCCC
GCGCCCCGTAAAAAGGGACGGCCCGCCCGAAGACCCCTAAACTCTGTTTTTAGTGGAACTTCTGAGTAAAACAAACAAAT
AAATCAAAACTTTCAACAACGGATCTCTTGGTTCTGGCATCGATGAAGAACGCAGCAAAATGCGATAAGTAATGTGAATT
GCAGAATTCAGTGAATCATCGAATCTTTGAACGCACATTGCGCCCGCCAGTATTCTGGCGGGCATGCCTGTTCGAGCGTC
ATTTCAACCCTCAAGCTCAGCTTGGTGTTGGGACTCGCGGTAACCCGCGTTCCCCAAATCGATTGGCGGTCACGTCGAGC
TTCCATAGCGTAGTAATCATACACCTCGTTACTGGTAATCGTCGCGGCCACGCCGTAAAACCCCAACTTCTGAATGTTGA
CCTCGGATCAGGTAGGAATACCCGCTGAACTTAAGCATATCAATAAGG
>F.equiseti_MW785181.1
ACAAGGTCTCCGTTGGTGAACCAGCGGAGGGATCATTACCGAGTTTACAACTCCCAAACCCCTGTGAACATACCTATACG
TTGCCTCGGCGGATCAGCCCGCGCCCCGTAAAACGGGACGGCCCGCCCGAGGACCCCTAAACTCTGTTTTTAGTGGAACT
TCTGAGTAAAACAAACAAATAAATCAAAACTTTCAACAACGGATCTCTTGGTTCTGGCATCGATGAAGAACGCAGCAAAA
TGCGATAAGTAATGTGAATTGCAGAATTCAGTGAATCATCGAATCTTTGAACGCACATTGCGCCCGCCAGTATTCTGGCG
GGCATGCCTGTTCGAGCGTCATTTCAACCCTCAAGCTCAGCTTGGTGTTGGGACTCGCGGTAACCCGCGTTCCCCAAATC
GATTGGCGGTCACGTCGAGCTTCCATAGCGTAGTAATCATACACCTCGTTACTGGTAATCGTCGCGGCCACGCCGTAAAA
CCCCAACTTCTGAATGTTGACCTCGGATCAGGTAGGAATACCCGCTGAACTTAAGCATATCAA
>F.delfinoides_OL376608.1
TACTCCGTAGGTGAACCTGCGGAGGGATCATTTCCGAGTTTACAACTCCCAAACCCCTGTGAACATACCTATAACGTTGC
CTCGGCGGCTCCCCGCCTCCCCGTAACACGGGAGCGGCCCGCCAGAGGACCCAACAAACTCTGTTATTTTCAGTATCTTC
TGAGTAAAAACACAATCAATTAAAACTTTCAACAACGGATCTCTTGGTTCTGGCATCGATGAAGAACGCAGCGAAATGCG
ATAAGTAATGTGAATTGCAGAATTCAGTGAATCATCGAATCTTTGAACGCACATTGCGCCCGCCAGTACTCTGGCGGGCA
TGCCTGTTCGAGCGTCATTACATCCCTCAAGCCCCAGCGGCTTGGTGTTGGGCTTCGGCCGTCCTCAGCGGCGGCCGTGC
CCCAAATACAGTGGCGGTCTCGCCCCCGGCTCCTCTGCGTAGTAGTAACA
>F.graminearum_MH054929.1
```

TTACCGAGTTTACAACTCCCAAACCCCTGTGAACATACCTTATGTTGCCTCGGCGGATCAGCCCGCGCCCCGTAAAAAGG
GACGGCCCGCCGCAGGAACCCTAAACTCTGTTTTTAGTGGAACTTCTGAGTATAAAAAACAAATAAATCAAAACTTTCAA
CAACGGATCTCTTGGTTCTGGCATCGATGAAGAACGCAGCAAAATGCGATAAGTAATGTGAATTGCAGAATTCAGTGAAT
CATCGAATCTTTGAACGCACATTGCGCCCGCCAGTATTCTGGCGGGCATGCCTGTTCGAGCGTCATTTCAACCCTCAAGC
CCAGCTTGGTGTTGGGAGCTGCAGTCCTGCTGCACTCCCCAAATACATTGGCGGTCACGTCGAGCTTCCATAGCGTAGTA
ATTTACACATCGTTACTGGTAATCGTCGCGGCCACGCCGTTAAACCCCAACTTCTGAATGTGACC
>F.solani_JQ625564.1
TAAGAGGAAGTAAAAGTCGTAACAAGGTTTCGTTGGTGAACCAGCGGAGGGATCATTACCGAGTTATACAACTCATCAAC
CCTGTGAACATACCTATAACGTTGCCTCGGCGGAACAGACGGCCCCGTAACACGGGCCGCCCCCGCCAGAGGACCCCCT
AACTCTGTTTCTATAATGTTTCTTCTGAGTAAACAAGCAAATAAATTAAAACTTTCAACAACGGATCTCTTGGCTCTGGC
ATCGATGAAGAACGCAGCGAAATGCGATAAGTAATGTGAATTGCAGAATTCAGTGAATCATCGAATCTTTGAACGCACAT
TGCGCCCGCCAGTATTCTGGCGGGCATGCCTGTTCGAGCGTCATTACAACCCTCAGGCCCCCGGGCCTGGCGTTGGGGAT
CGGCGGAAGCCCCCTGCGGGCACAACGCCGTCCCCCAAATACAGTGGCGGTCCCGCCGCAGCTTCCATTGCGTAGTAGCT
AACACCTCGCAACTGGAGAGCGGCGCGGCCACGCCGTAAAACACCCAACTTCTGAATGTTGACCTCGAATCAGGTAGGAA
TACCCGCTGAACTTATGCATATCAATAAGCGGAGGAAAAGAAACCAACAGGGATTGCCCCAGTA
>F.verticillioides_KT357570.1
TGCGGAGGGATCATTACCGAGTTTACAACTCCCAAACCCCTGTGAACATACCAATTGTTGCCTCGGCGGATCAGCCCGCT
CCCGGTAAAACGGGACGGCCCGCCAGAGGACCCCTAAACTCTGTTTCTATATGTAACTTCTGAGTAAAACCATAAATAAA
TCAAAACTTTCAACAACGGATCTCTTGGTTCTGGCATCGATGAAGAACGCAGCAAAATGCGATAAGTAATGTGAATTGCA
GAATTCAGTGAATCATCGAATCTTTGAACGCACATTGCGCCCGCCAGTATTCTGGCGGGCATGCCTGTTCGAGCGTCATT
TCAACCCTCAAGCCCAGCTTGGTGTTGGGACTCGCGAGTCAAATCGCGTTCCCCAAATTGATTGGCGGTCACGTCGAGCT
TCCATAGCGTAGTAGTAAAACCCTCGTTACTGGTAATCGTCGCGGCCACGCCGTTAACCCCAACTTCTGAATGTGACCTC
GATCA
>F.subglutinans_OM185557.1
AGGTCTCCGTTGGTGAACCAGCGGAGGGATCATTACCGAGTTTACAACTCCCAAACCCCTGTGAACATACCAATTGTTGC
CTCGGCGGATCAGCCCGCTCCCGGTAAAACGGGACGGCCCGCCAGAGGACCCCTAAACTCTGTTTCTATATGTAACTTCT
GAGTAAAACCATAAATAAATCAAAACTTTCAACAACGGATCTCTTGGTTCTGGCATCGATGAAGAACGCAGCAAAATGCG
ATAAGTAATGTGAATTGCAGAATTCAGTGAATCATCGAATCTTTGAACGCACATTGCGCCCGCCAGTATTCTGGCGGGCA
TGCCTGTTCGAGCGTCATTTCAACCCTCAAGCCCAGCTTGGTGTTGGGACTCGCGAGTCAAATCGCGTTCCCCAAATTGA
TTGGCGGTCACGTCGAGCTTCCATAGCGTAGTAGTAAAACCCTCGTTACTGGTAATCGTCGCGGCCACGCCGTTAAACCC
CAACTTCTGAATGTTGACCTCGGATCAGGTAGGAATACCCGCTGAACT
>F.proliferatum_MW704332.1
TGGAAGTAAAAAATCGTAACAAGGTCTCCGTTGGTGAACCAGCGGAGGGATCATTACCGAGTTTACAACTCCCAAACCCC
TGTGAACATACCAATTGTTGCCTCGGCGGATCAGCCCGCTCCCGGTAAAACGGGACGGCCCGCCAGAGGACCCCTAAACT
CTGTTTCTATATGTAACTTCTGAGTAAAACCATAAATAAATCAAAACTTTCAACAACGGATCTCTTGGTTCTGGCATCGA
TGAAGAACGCAGCAAAATGCGATAAGTAATGTGAATTGCAGAATTCAGTGAATCATCGAATCTTTGAACGCACATTGCGC
CCGCCAGTATTCTGGCGGGCATGCCTGTTCGAGCGTCATTTCAACCCTCAAGCCCAGCTTGGTGTTGGGACTCGCGAGTC
AAATCGCGTTCCCCAAATTGATTGGCGGTCACGTCGAGCTTCCATAGCGTAGTAGTAAAACCCTCGTTACTGGTAATCGT
CGCGGCCACGCCGTTAAACCCCAACTTCTGAATGTTGACCTCGGATCAGGTAGGAATACCCGCTGAACTTAAGCATATCA
AAA
>F.sacchari_OL347721.1
TACCGAGTTTACAACTCCCAAACCCCTGTGAACATACCAATTGTTGCCTCGGCGGATCAGCCCGCTCCCGGTAAAACGGG
ACGGCCCGCCAGAGGACCCCCAAACTCTGTTTCTATATGTAACTTCTGAGTAAAACCATAAATAAATCAAAACTTTCAAC
AACGGATCTCTTGGTTCTGGCATCGATGAAGAACGCAGCAAAATGCGATAAGTAATGTGAATTGCAGAATTCAGTGAATC
ATCGAATCTTTGAACGCACATTGCGCCCGCCAGTATTCTGGCGGGCATGCCTGTTCGAGCGTCATTTCAACCCTCAAGCC
CAGCTTGGTGTTGGGACTCGCGAGTCAAATCGCGTTCCCCAAATTGATTGGCGGTCACGTCGAGCTTCCATAGCGTAGTA
GTAAAACCCTCGTTACTGGTAATCGTCGCGGCCACGCCGTTAAACCCCAACTTCTGAATGTTGACCTCGGATCAGGTAGG
AATACCCGCTGAACTTAAGCATAT
>F.clavum_MZ890488.1
AACAAGGTCTCCGTTGGTGAACCAGCGGAGGGATCATTACCGAGTTTACAACTCCCAAACCCCTGTGAACATACCTATAC
GTTGCCTCGGCGGATCAGCCCGCGCCCTGTAAAAAGGGACGGCCCGCCCGAGGACCCTAAACTCTGTTTTTAGTGGAACT
TCTGAGTAAAACAAACAAATAAATCAAAACTTTCAACAACGGATCTCTTGGTTCTGGCATCGATGAAGAACGCAGCAAAA
TGCGATAAGTAATGTGAATTGCAGAATTCAGTGAATCATCGAATCTTTGAACGCACATTGCGCCCGCCAGTATTCTGGCG
GGCATGCCTGTTCGAGCGTCATTTCAACCCTCAAGCTCAGCTTGGTGTTGGGACTCGCGGTAACCCGCGTTCCCCAAATC

GATTGGCGGTCACGTCGAGCTTCCATAGCGTAGTAATCATACACCTCGTTACTGGTAATCGTCGCGGCCACGCCGTAAAA
CCCCAACTTCTGAATGTTGACCTCGGATCAGGTAGGAATACCCGCTGAACTTAAGCATAT
>Setophoma_terrestres_OL960208.1
GGATCATTATCAAAAGTCAAGTCGGGGGCTGTAAAGCTCTCGTCTACACCCATGTCTTTTGCGTACTCTTGTTTCCTCGG
TGGCGCAAGCTGCCGATTGGACAAACCAAAACCTTTTTTGTAATTGCAATCAGCGTCTGAAAATAATCTAATTATTTACA
ACTTTCAACAACGGATCTCTTGGTTCTGGCATCGATGAAGAACGCAGCGAAATGCGATAAGTAGTGTGAATTGCAGAATT
CAGTGAATCATCGAATCTTTGAACGCACATTGCGCCCCTTGGTATTCCATGGGGCATGCCTGTTCGAGCGTCATTTGTAC
CCTCAAGCTTTGCTTGGTGTTGGGCGTCTTGTCGTATTACGACTCGCCTTAAATTCATTGGCAGCCGGCACTTTGGCCTA
GGAGCGCAGCACATTTTGCGATCGTAGCCCGTTGTACTGGCGTCCATCAAGAACATTTACCACGTTTGACCTCGGATCAG
GTAGGGATACCCGCTGAA
>Alternaria_solani_OK427286.1
TCCGTAGGTGAACCTGCGGAGGGATCATTACACAAATATGAAGGCGGGCTGGCACCTCCCGGGGTGGCCAGCCTTGCTGA
ATTATTTCACCCGTGTCTTTTGCGTACTTCTTGTTTCCTTGGTGGGCTCGCCCACCACAAGGACCAACCCATAAACCTTT
TTGCAATGGCAATCAGCGTCAGTAACAATGTAATAATTTACAACTTTCAACAACGGATCTCTTGGTTCTGGCACCGATGA
AGAACGCAGCGAAATGCGATAAGTAGTGTGAATTGCAGAATTCAGTGAATCATCGAATCTTTGAACGCACATTGCGCCCT
TTGGTATTCCAAAGGGCATGCCTGTTCGAGCGTCATTTGTACCCTCAAGCTTTGCTTGGTGTTGGGCGTCTTTTTGTCTC
CCCTTGCGGGAGACTCGCCTTAAAGTCATTGGCAGCCGGCCTACTGGTTTCGGAGCGCAGCACAAGTCGCGCTCTCTTCC
AGCCCCAAGGTCTAGCATCCACCAAGCCTTTTTTTTCAACTTTTGACCTCGGATCAGGTAGGGATACCCGCTGAACTTAA
GCATATCAATAAGCGGAGGA
>Botrytis_cinérea_MW301135.1
GGAAGTAAAAGTCGTAACAAGGTTTCCGTAGGTGAACCTGCGGAAGGATCATTACAGAGTTCATGCCCGAAAGGGTAGAC
CTCCCACCCTTGTGTATTATTACTTTGTTGCTTTGGCGAGCTGCCTTCGGGCCTTGTATGCTCGCCAGAGAATACCAAAA
CTCTTTTTATTAATGTCGTCTGAGTACTATATAATAGTTAAAACTTTCAACAACGGATCTCTTGGTTCTGGCATCGATGA
AGAACGCAGCGAAATGCGATAAGTAATGTGAATTGCAGAATTCAGTGAATCATCGAATCTTTGAACGCACATTGCGCCCC
TTGGTATTCCGGGGGGCATGCCTGTTCGAGCGTCATTTCAACCCTCAAGCTTAGCTTGGTATTGAGTCTATGTCAGTAAT
GGCAGGCTCTAAAATCAGTGGCGGCGCCGCTGGGTCCTGAACGTAGTAATATCTCTCGTTACAGGTTCTCGGTGTGCTTC
TGCCAAAACCCAAATTTTTCTATGGTTGACCTCGGATCAGGTAGGGATACCCGCTGAACTTAAGCATATCA
>Passalora_fulva_KM488557.1
TCTCCGTAGGTGAACCTGCGGAGGGATCATTACTGAGTGAGGGCTCACGCCCGACCTCCAACCCTCTGTGAACCAACTCT
GTTGCTTCGGGGGCGACCCCGCCGTTTCGGCGACGGCGCCCCCGGAGATCATCAAACACTGCATCTTTGCGTCGGAGTCT
TAAAGTAAATTTAAACAAAACTTTCAACAACGGATCTCTTGGTTCTGGCATCGATGAAGAACGCAGCGAAATGCGATAAG
TAATGTGAATTGCAGAATTCAGTGAATCATCGAATCTTTGAACGCACATTGCGCCCCGTGGTATTCCGCGGGGCATGCCT
GTTCGAGCGTCATTTCACCACTCAAGCCTAGCTTGGTATTGGGCGTCGCGGTTCCGCGCGCCTTGAAGTCTCCGGCTGAG
CAGTTCGTCTCTAAGCGTTGTGGCATATATTTCGCTAAAGAGTTCGGGCGGCTTTTGGCCGTTAAATCTTTTCAAAGGTT
GACCTCGGATCAGGTAGGGATACCCGCTGAACTTAAGCATATCA
>Colletotrichum_coccodes_KF494014.1
TGCTCTATACCCTTTGTGACATACCTAACTGTTGCTTCGGCGGGCAGGGGGTGCCGCCTGCGGACCCCCCTCCCGGCCCT
GCCCTCACGGGCGGAGCGCCCGCCGGAGGATACCAAACTCTATTTTAACGACGTTTCTTCTGAGTGGCACAAGCAAATAA
TTAAAACTTTCAACAACGGATCTCTTGGTTCTGGCATCGATGAAGAACGCAGCGAAATGCGATAAGTAATGTGAATTGCA
GAATTCAGTGAATCATCGAATCTTTGAACGCACATTGCGCCCGCCAGCATTCTGGCGGGCATGCCTGTTCGAGCGTCATT
TCAACCCTCAAGCTCTGCTTGGTGTTGGGGCCCTACGGTTGACGTAGGCCCTTAAAGGTAGTGGCGGACCCTCTCGGAGC
CTCCTTTGCGTAGTAACTAACGTCTCGCACTGGGATTCGGAGGGACTCTTGCCGTAAAACCCCCAAATTTTTAAAGGTTG
ACCTCGGATCAGGTAGGAATACCCGCTGAACTTAAGCATATCAATAAGCGGAGGA
>Leveillula_taurica_OK036584.1
AGAGTGGCGACACTCCCCCAGGGCCGGAAAGTTGTCCAAACTTGGTCATTTAGAGGAAGTAAAAGTCGTAACAAGGTTTC
CGTAGGTGAACCTGCGGAAGGATCATTACAGAGCGTGAAGACCTCGGCCCCTCCACAGCGCAAGCTGGTGCGAGGGACAC
ATGCCGGGGTCGACCCTCCCACCCGTGTCGACTCGTCTCCTGTTGCTTTGGCAGGCCGACTGCCTAGCGGTCCTCTGGCT
CTCGGGCTGGAGTGCGCCTGCCAGAGACTATTCAACTCGTGTTCTGGATGAAGTCTGAGCAATCAAGCAATAAAAATGAA
TAAGTTAAAACTTTCAACAACGGATCTCTTGGCTCTGGCATCGATGAAGAACGCAGCGAAATGCGATAAGTAATGTGAAT
TGCAGAATTTAGTGAATCATCGAATCTTTGAACGCACATTGCGCCCCTGGGTACTCCTAGGGGCATGCCTGTTCGAGCGT
CATAACAACCCGTCGAGCCGACTAGGCTTGGTCTTGGGGCTCGCCCGCATTTGGCGCGGCGGCTCTTAAACGCAGTGGCG
GTGCCGGTGGTGCTTTCCGCGTAGTCACATTTCTCGCGCGAGGGCAGAATCCGGACCCAGCCAGCAACCACAAAGTCCGC
AGCGCTCTGCGCGGCGACTTTTGTACTTCTTCTGGTTGACCTCGAATCAGGTAGGGATACCCGCTGAACTTAAGCATATC
AATAAG

>Oidium_lycopersici_AF229019.1
ATCATTACAGAGCGTGAGGCTCAGTCGTGGCGTCAGCTGCGTGCTGGGCCGACCCTCCCACCCGTGTCGATTTCTATCTT
GTTGCTTTGGCGGGCCGGGCTACGTCGTCGCTGCCCGTACGGACATGTGTCGGCCGCCCACCGGTTTCGACTGGAGCGCG
TCCGCCAAAGACCTAACCAAAACTCATGTTGTCTTTGTCGTCTCAGCTTTATTATTGAATTGATAAAACTTTCAACAACG
GATCTCTTGGCTCTGGCATCGATGAAGAACGCAGCGAAATGCGATAAGTAATGTGAATTGCAGAATTTAGTGAATCATCG
AATCTTTGAACGCACATTGCGCCCCTTGGTATTCCGAGGGGCATGCCTGTTCGAGCGTCATAACACCCCCTCCAGCTGCC
TTTGTGTGGTTGCGGTGTTGGGGCCCGTCGCGTTGCGGCAGCTCTTAAAGATAGTGGCGGTCCTGGCGTGGGCTCTACGC
GTAGTAACTTGCTTCTCGCGACAGAGTGACGACAGTGGCTTGCCAAAAGCCCGTTTGTTCCAGTCACATGGATCACAGGT
>Pseudopyrenochaeta_neolycopersici_MG846017.1
TCCGTAGGTGAACCTGCGGAAGGATCATTACAGAGCGTGAGGCTCAGTCGTGGCGTCAGCTGCGTGCTGGGCCGACCCTC
CCACCCGTGTCGATTTCTATCTTGTTGCTTTGGCGGGCCGGGCTACGTCGTCGCTGCCCGTACGGACATGTGTCGGCCGC
CCACCGGTTTCGACTGGAGCGCGTCCGCCAAAGACCTAACCAAAACTCATGTTGTCTTTGTCGTCTCAGCTTTATTATTG
AATTGATAAAACTTTCAACAACGGATCTCTTGGCTCTGGCATCGATGAAGAACGCAGCGAAATGCGATAAGTAATGTGAA
TTGCAGAATTTAGTGAATCATCGAATCTTTGAACGCACATTGCGCCCCTTGGTATTCCGAGGGGCATGCCTGTTCGAGCG
TCATAACACCCCCTCCAGCTGCCTTTGTGTGGTTGCGGTGTTGGGGCCCGTCGCGTTGCGGCAGCTCTTAAAGATAGTGG
CGGTCCTGGCGTGGGCTCTACGCGTAGTAACTTGCTTCTCGCGACAGAGTGACGACAGTGGCTTGCCAAAAGCCCGTTTG
TTCCAGTCACATGGATCACAGGTTGACCTCGAATCAGGTAGGAATACCCGCTGAACTTAAGCATATCAATAAGCGGAG
>Pseudopyrenochaeta_lycopersici_MK052946.1
TAACAAGGTTTCCGTAGGTGAACCTGCGGAAGGATCATTAACTGTAACATTGGGGGCTGGTGGAGGGTTGCGCACTTTGT
GCGTGTTCCTTCCCCGCCCTGTCTGCTACTGCCCATGTCTTTTGCGTACCCATTGTTTCCTCGGCGGGTTTGCCCGTCGA
TTGGACACTACAACCCTTTGTAATTGCAATCAGCGTCAGAAAACTATAATTATTACAACTTTCAACAACGGATCTCTTGG
TTCTGGCATCGATGAAGAACGCAGCGAAATGCGATAAGTAGTGTGAATTGCAGAATTCAGTGAATCATCGAATCTTTGAA
CGCACATTGCGCCCCTTGGTATTCCATGGGGCATGCCTGTTCGAGCGTCATTTGTACCCTCAAGCATTGCTTGGTGTTGG
GTGTTTGTCCCGCTGTTACGCGTGGACTCGCCTTGAAGCAATTGGCAGCCGGCAATCTGGTGATGGAGCGCAGCACATTT
TGCGCTTCTTGCTATAGATAACGGCGTCCATCAAGCCTTTTTTTTGCTCTTGACCTCGGATCAGGTAGGGATACCCGCTG
AACTTAAGCATATC
>Pyrenochaeta_lycopersici_AM944362.1
TCCGTAGGTGAACCTGCGGAAGGATCATTAACTGTATTACGGGGGGCCGGCGTGGGATTGCGTGCTTTGGTGCGCCTTCC
CTCCCCGCCCTGTCTGATACTACCCGTGTCTTTTGCGTACCAATTGTTTCCTCGGTAGGCTTGCCTGCCGGCCGGACACC
ATAAAACCTTTTGTGATTGCAGTCAGCGTCAGAAAACTACAATAATTACAACTTTCAACAACGGATCTCTTGGTTCTGGC
ATCGATGAAGAACGCAGCGAAATGCGATAAGTAGTGTGAATTGCAGAATTCAGTGAATCATCGAATCTTTGAACGCACAT
TGCGCCCCTTGGTATTCCACGGGGCATGCCTGTTCGAGCGTCATTTGTACCCTCAAGCTCTGCTTGGTGTTGGGTGTTTG
TCCCGCTTTGCGCGTGGACTCGCCTCAAAGCAATTGGCAGCCGGCAATCTGGTTATAGAGCGCAGCACATTTTGCGCTTC
TTGCCACGGATGTCGGCGTCCATCAAGCCTACACTTTTGCTCTTGACCTCGGATCAGGTAGGGATACCCGCTGAACTTAA
GCATATCAATAAGCGGAGAATCGA
>Rhizoctonia_solani_MW498395.1
GACCTGCGGAAGGATCATTATTGAATTTAATGTAGAGTTGGTTGTAGCTGGCCTAATAAATTAATGTTGGGCATGTGCAC
ACCTTCTCTTTCATCCACACACACCTGTGCACCTGTGAGACAGTAGGGGATTTTTAATTTAATTTAATTGGACCCTCTGT
CTACTTAATTCATATAAAATCAATTTAATTAAAAATGAATGTAATTGATGTAACGCATCTAATACTAAGTTTCAACAACG
GATCTCTTGGCTCTCGCATCGATGAAGAACGCAGCGAAATGCGATAAGTAATGTGAATTGCAGAATTCAGTGAATCATCG
AATCTTTGAACGCACCTTGCGCTCCTTGGTATTCCTTGGAGCATGCCTGTTTGAGTATCATGAAATCTTCAAAGTAAATC
TTTTGTTAATTCAATTGGTTCTGCTTTGGTATTGGAGGTTATTGCAGCTTCACACCTGCTCCTCTTTGTGCATTAGCTGG
ATCTCAGTGTTATGCTTGGTTCCACTCAGCGTGATAAGTATCTATCGCTGAGGACACTGTAACAGGTGGCCAAGGTAAAT
GCAGATGAACCGCTTCTAATAGTCCATTAATTTGGACAATATTTTTATGATCTGATCTCAAATCAGGTAGGACTACCCGC
TGAACTTAAGCATATCAATAAGCCGGAGGGAAGGATAATTATTGAATTTAATGTAAAGTTGGTTGTAGCTGGCCAAATAA
AT
>Septoria_lycopersici_KF251463.1
GCGGAGGGATCATTACTGAGTGAGGGCCTTCGGGCTCGACCTCCAACCCTTTGTGAACACAACTTGTTGCTTCGGGGGCG
ACCCTGCCGTTTCGACGGCGAGCGCCCCCGGAGACCTTCAAACACTGCATCTTTGCGTCGGAGTTTAAGTAAATTAAACA
AAACTTTCAACAACGGATCTCTTGGTTCTGGCATCGATGAAGAACGCAGCGAAATGCGATAAGTAATGTGAATTGCAGAA
TTCAGTGAATCATCGAATCTTTGAACGCACATTGCGCCCTTTGGTATTCCGAAGGGCATGCCTGTTCGAGCGTCATTTCA
CCACTCAAGCCTGGCTTGGTATTGGGCGCCGCGGTCAATCCGCGCGCCTCAAAGTCTCCGGCTGAGCTGTCCGTCTCTAA
GCGTTGTGATTTCATTAATCGCTTCGGAGTGCGGGCGGCCGCGGCCGTTAAATCTTTCACAAGGTTGACCTCGGATCAGG
TAGGGATACCCGCTG

>Sclerotinia_sclerotiorum_MF563992.1
GGGCTCGTACTCTGCCAGAAGGTAGACCTCCCACCCTTGTGTATTATTACTTTGTTGCTTTGGCGAGCTGCTCTTCGGGG
CCTTGTATGCTCGCCAGAGAATATCAAAACTCTTTTTATTAATGTCGTCTGAGTACTATATAATAGTTAAAACTTTCAAC
AACGGATCTCTTGGTTCTGGCATCGATGAAGAACGCAGCGAAATGCGATAAGTAATGTGAATTGCAGAATTCAGTGAATC
ATCGAATCTTTGAACGCACATTGCGCCCCTTGGTATTCCGGGGGGCATGCCTGTTCGAGCGTCATTTCAACCCTCAAGCT
CAGCTTGGTATTGAGTCCATGTCAGTAATGGCAGGCTCTAAAATCAGTGGCGGCGCCGCTGGGTCCTGAACGTAGTAATA
TCTCTCGTTACAGGTTCTCGGTGTGCTTCTGCCAAAACCCAAATTTTCTATGGTTGACCTCGGATCAGGTAGGGATACCC
GCTGAACTTAAGCATATCAATAAGCGGAGGAA
>Athelia_rolfsii_MW349665.1
CCCTTCCGTAGGGAACCTTGGGGAAGGTCCATTATGGAATTCATATATGGGAGGAAGTGGTGTTGGTAAGAAATATGGCA
GGTCCCCCTTCGGGGGCTATATAATATATCCCCCTGTGACCCACCGGTAGTCAGGAGAATTCTTAATTAGGATCCCCTAA
AATAATTTTTATGGAAGGTTCCATAGAAGGTTTTCATATGGAACCTTTGTTTTCGGCCAAGTTTTTTTAAATAAAAAAAT
ATACAACTTTCAACAACGGATCTCTTGGCTCTTGCATCGATGAAGAACGCAGCGAAATGCGATAAGTAATGTGAATTGCA
GAATCCAGTGAATCATCGAATCTTTGAACGCACCTTGCGCCCTTTGGTATTCCGAGGGGCATGCCTGTTTGAGAGTCATT
AAATTCTCAACCTTACAAATTTTTGTATTTGTCAAGGCTTGGATGTGAGAGTTGCTGGTTAGAGTATATTCTGACTGGCT
CTCTTTAAAACTATTAGTAGGACATGTAGAAATGCCTACGGTTGGTGTGATAATATGTCTACGCCTATACCGGAAGGGGA
TTCTAGCTTGTATGTACTACTTATAAAATCATGCGCATATATCTAGCATATAAGTGCATATATTGACCATTTGACCTCAA
ATCAGGTAGGACTACCCGCTGAA
>Stemphylium_vesicarium_MZ099818.1
GAGGGCTCCAGCTTGTCTGAATTATTCACCCATGTCTTTTGCGCACTTCTTGTTTCCTGGGCGGGTTCGCCCGCCACCAG
GACCAAACCATAAACCTTTTTGTAATTGCAATCAGCGTCAGTAAACAATGTAATTATTACAACTTTCAACAACGGATCTC
TTGGTTCTGGCATCGATGAAGAACGCAGCGAAATGCGATACGTAGTGTGAATTGCAGAATTCAGTGAATCATCGAATCTT
TGAACGCACATTGCGCCCTTTGGTATTCCAAAGGGCATGCCTGTTCGAGCGTCATTTGTACCCTCAAGCTTTGCTTGGTG
TTGGGCGTCTTTGTCTCTCACGAGACTCGCCTTAAAATGATTGGCAGCCGACCTACTGGTTTCGGAGCGCAGCACAATTC
TTGCACTTTGAATCAGCCTTGGTTGAGCATCCATCAAGACCACATTTTTTTCAACTTTTGACCTCGGATCAGGTAGGGAT
ACC
>Stemphylium_lycopersici_MZ093130.1
GAGGGCTCCAGCTTGTCTGAATTATTCACCCATGTCTTTTGCGCACTTCTTGTTTCCTGGGCGGGTTCGCCCGCCACCAG
GACCAAACCATAAACCTTTTTGTAATTGCAATCAGCGTCAGTAAACAATGTAATTATTACAACTTTCAACAACGGATCTC
TTGGTTCTGGCATCGATGAAGAACGCAGCGAAATGCGATACGTAGTGTGAATTGCAGAATTCAGTGAATCATCGAATCTT
TGAACGCACATTGCGCCCTTTGGTATTCCAAAGGGCATGCCTGTTCGAGCGTCATTTGTACCCTCAAGCTTTGCTTGGTG
TTGGGCGTCTTGTCTCTCACGAGACTCGCCTTAAAATCATTGGCAGCCGACCTACTGGTTTCGGAGCGCAGCACAATTCT
TGCACTTTGAATCAGCCTTGGTTGAGCATCCATCAAGACCCTATTTTTTTTAACTTTTGACCTCGGATCAGGTAGGGATA
CC
>Stemphylium_eturmiunum_MZ093121.1
GAGGGCTCCAGCTTGTCTGAATTATTCACCCATGTCTTTTGCGCACTTCTTGTTTCCTGGGCGGGTTCGCCCGCCACCAG
GACCAAACCATAAACCTTTTTGTAATTGCAATCAGCGTCAGTAAACAATGTAATTATTACAACTTTCAACAACGGATCTC
TTGGTTCTGGCATCGATGAAGAACGCAGCGAAATGCGATACGTAGTGTGAATTGCAGAATTCAGTGAATCATCGAATCTT
TGAACGCACATTGCGCCCTTTGGTATTCCAAAGGGCATGCCTGTTCGAGCGTCATTTGTACCCTCAAGCTTTGCTTGGTG
TTGGGCGTCTTTGTCTCTCACGAGACTCGCCTTAAAATGATTGGCAGCCGACCTACTGGTTTCGGAGCGCAGCACAATTC
TTGCACTTTGAATCAGCCTTGGTTGAGCATCCATCAAGACCACATTTTTTAACTTTTGACCTCGGATCAGGTAGGGATAC
C
>Verticillium_dahliae_GU060637.1
GGAAGTAAAAGTCGTAACAAGGTCTCCGTTGGTGAACCAGCGGAGGGATCATTACCGAGTATCTACTCATAACCCTTTGT
GAACCATATTGTTGCTTCGGCGGCTCGTTCTGCGAGCCCGCCGGTCCATCAGTCTCTCTGTTTATACCAACGATACTTCT
GAGTGTTCTTAGCGAACTATTAAAACTTTTAACAACGGATCTCTTGGCTCTAGCATCGATGAAGAACGCAGCGAAACGCG
ATATGTAGTGTGAATTGCAGAATTCAGTGAATCATCGAATCTTTGAACGCACATGGCGCCTTCCAGTATCCTGGGAGGCA
TGCCTGTCCGAGCGTCGTTTCAACCCTCGAGCCCCAGTGGCCCGGTGTTGGGGATCTACGTCTGTAGGCCCTTAAAAGCA
GTGGCGGACCCGCGTGGCCCTTCATTGCGTAGTAGTTACAGCTCGCATCGGAGTCCCGCAGGCGCTTGCCTCTAAACCCC
CTACAAGCCCGCCTCGTGCGGCAACGGTTGACCTCGGATCAGGTAGGAATACCCGCTGAACTTAAGCATATCAATAAGCG
GAGGA
