# Supplementary material for: A TaqMan® Assay Allows an Accurate Detection and Quantification of Fusarium spp., the Causal Agents of Tomato Wilt and Rot Diseases
Source: Biology (Basel). 2023 Feb 8;12(2):268. doi: 10.3390/biology12020268 (PMC9953614; doi:10.3390/biology12020268)
Supplement: Supplementary file 1 [file biology-12-00268-s001.zip › Figure S2.pdf]

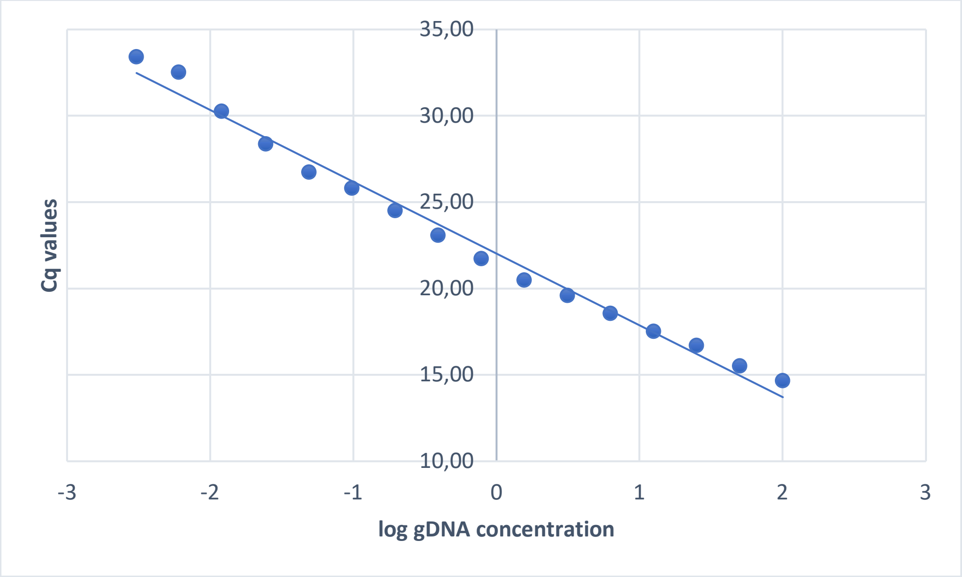

Figure S2: Sensitivity and linearity of the *Fusarium* spp.-specific qPCR assay. Standard curve constructed based on Cq values obtained from the log of two-fold dilution series of gDNA from *F. oxyporum* f. sp *radicis-lycopersici*. gDNA concentrations vary from 100 ng to 3.05 x 10^{-3} ng.
