# Supplementary material for: A TaqMan® Assay Allows an Accurate Detection and Quantification of Fusarium spp., the Causal Agents of Tomato Wilt and Rot Diseases
Source: Biology (Basel). 2023 Feb 8;12(2):268. doi: 10.3390/biology12020268 (PMC9953614; doi:10.3390/biology12020268)
Supplement: Supplementary file 1 [file biology-12-00268-s001.zip › Table S1.pdf]

Table S1 Quantification of *Fusarium* spp. in tomato field and control samples using the *Fusarium* spp.-specific qPCR assay. Data are expressed as quantification cycle (Cq) values.

Field Samples ID	Cq value
T1_1	N.D.
T1_2	N.D.
T1_3	N.D.
T1_4	23,94
T1_5	N.D.
T1_6	N.D.
T1_7	23,50
T1_8	N.D.
T1_9	26,35
T1_10	22,28
T1_11	27,88
T1_12	27,90
T1_13	17,25
T1_14	N.D.
T1_15	N.D.
T2_1	N.D.
T2_2	N.D.
T2_3	N.D.
T2_4	N.D.
T2_5	N.D.
T2_6	N.D.
T2_7	27,11
T2_8	N.D.
T2_9	N.D.
T2_10	22,13
T2_11	23,24
T2_12	N.D.
T2_13	28,26
T2_14	N.D.
T2_15	27,59

Control Samples ID	Cq value
T1_C1	N.D.
T2_C2	N.D.
T1_C3	N.D.
T1_C4	24,65
T1_C5	N.D.
T1_C6	N.D.
T1_C7	22,49
T1_C8	26,40
T2_C1	24,48
T2_C2	23,30
T2_C3	N.D.
T2_C4	N.D.
T2_C5	24,14
T2_C6	28,16
T2_C7	24,09
T2_C8	N.D.
